# Adsorption-Driven Symmetry Lowering in Single Molecules Revealed by Ångstrom-Scale Tip-Enhanced Raman Imaging

**DOI:** 10.1021/jacs.5c18593

**Published:** 2026-02-27

**Authors:** Rodrigo Cezar de Campos Ferreira, Borja Cirera, Jiří Doležal, Álvaro Gallego de Roa, Amandeep Sagwal, Petr Kahan, Rubén Canales, Fernando Aguilar-Galindo, Martin Švec, Pablo Merino

**Affiliations:** 1 Institute of Organic Chemistry and Biochemistry, Czech Academy of Sciences, CZ-160 00 Praha 6, Czech Republic; 2 Institute of Physics, 86889 Czech Academy of Sciences, CZ-162 00 Praha 6, Czech Republic; 3 69570 Instituto de Ciencia de Materiales de Madrid (ICMM-CSIC), ES-28049 Madrid, Spain; 4 Departamento de Química, 16722 Universidad Autónoma de Madrid, ES-28049 Madrid,Spain; 5 Faculty of Mathematics and Physics, Charles University, Ke Karlovu 3, CZ-121 16 Praha 2, Czech Republic; 6 Institute for Advanced Research in Chemical Sciences (IAdChem), Universidad Autónoma de Madrid, ES-28049 Madrid, Spain

## Abstract

The vibrational landscape of adsorbed molecules is central to understanding surface interactions at the atomic scale, influencing phenomena from catalysis to molecular electronics. Recent advances in atomic-scale tip-enhanced Raman spectroscopy (TERS) have enabled vibrational mapping of single molecules with subnanometer spatial resolution, providing unprecedented insights into molecule–surface interactions by confining light in plasmonic picocavities. Here, we exploit TERS in a cryogenic scanning tunneling microscope junction to perform Raman hyperspectral mapping of single iron phthalocyanine (FePc) molecules in three nonequivalent adsorption configurations on Ag surfaces. We explore the changes in the vibrational modes of FePc molecules adsorbed on two distinct silver crystal terminations with differing symmetry, Ag(111) and Ag(110), revealing how subtle variations in the adsorption geometry due to substrate anisotropy can strongly influence molecular vibrations, lifting the degeneracy of individual normal modes. Our findings not only demonstrate the first use of subnanometer TERS mapping across different symmetry configurations but also provide a deeper understanding of how site-specific vibrational properties are intimately linked to local atomic environments. This capability paves the way for precisely tailoring surface interactions and controlling chemical reactions on the atomic scale.

## Graphical abstract

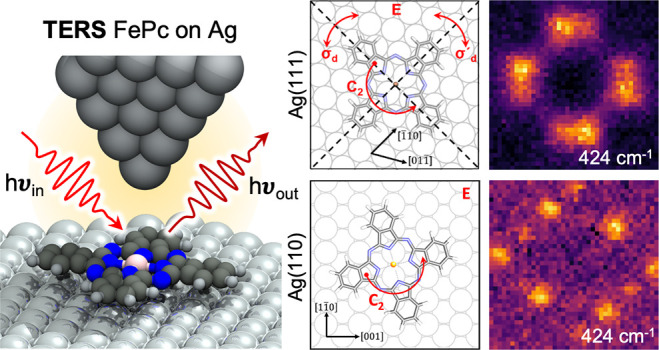

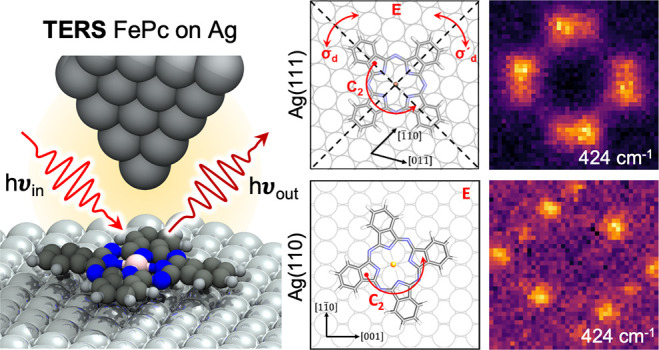

## Introduction

Molecular symmetry imposes strict selection rules on optical transitions because the interaction between light and matter must obey the symmetry of the molecule’s electronic, vibrational, and rotational states.^1^ In particular, Raman activity depends on how the polarizability tensor changes during a vibration; a vibrational mode is Raman active if its irreducible representation matches a linear combination of the polarizability tensor components for the molecule’s point group. However, exposing molecules to specific local nanoenvironments can result in atomic structure relaxation, leading to reduced symmetry.^2,3^ This can have a profound effect on the scattering activities of particular vibrational modes, which may become partially allowed, forbidden, or nondegenerate.^4^ Currently, very little is known about the effect of atomic deformations on Raman activity at the scale of single molecules.^5,6^

Tip-enhanced Raman spectroscopy (TERS) in cryogenic conditions within atomically defined plasmonic picocavities^7^ has recently demonstrated the ability to visualize individual vibrational modes in real space with submolecular resolution.^8–11^ With this technique, it is possible to reconstruct the chemical structure of single molecular adsorbates, reaching a single-bond limit,^12–14^ track formation and breaking of individual chemical bonds in real time,^15,16^ or even sequence fragments of single DNA strands.^17,18^ TERS also permits to perform correlative measurements that cannot be tackled with conventional surface-enhanced Raman spectroscopy, e.g., it allows exploring the links between the electronic configurations of individual adsorbates and their Raman spectra upon precise molecular nanomanipulations^19^ or controlling vibrations with time-resolved schemes at fs temporal resolution.^20^ In addition, evidence from STM experiments shows that single molecules on metal surfaces can undergo structural modifications^21,22^ and Jahn–Teller distortions upon molecular adsorption and concomitant spontaneous charging.^23,24^ However, the role of distortions in atomic-scale interactions across different interfaces has not been addressed in TERS experiments with submolecular resolution.

Here, we explore the breakdown of the D_4h_ symmetry of iron phthalocyanine (FePc) upon adsorption on the Ag(111) and Ag(110) surfaces using hyperspectral TERS mapping (schematically shown in Figure 1b and described in methods and Figure S1). We study three distinct adsorption configurations with progressively lowered C_2d_, C_2v_, and C_2_ symmetries (C_2d_ being a nonequivalent C_2v_ symmetry with the mirror planes lying on the diagonals of the molecule). We directly observe the symmetry reduction of the vibrations in the real-space-resolved TERS intensity patterns, evidencing the degeneracy lifting in the individual normal modes. We confront our experimental findings with density functional theory (DFT) calculations and corroborate the link between the molecular adsorption geometry, reduction of the symmetry, and the consequent alterations of the spatiospectral Raman fingerprints.

**Figure 1:**
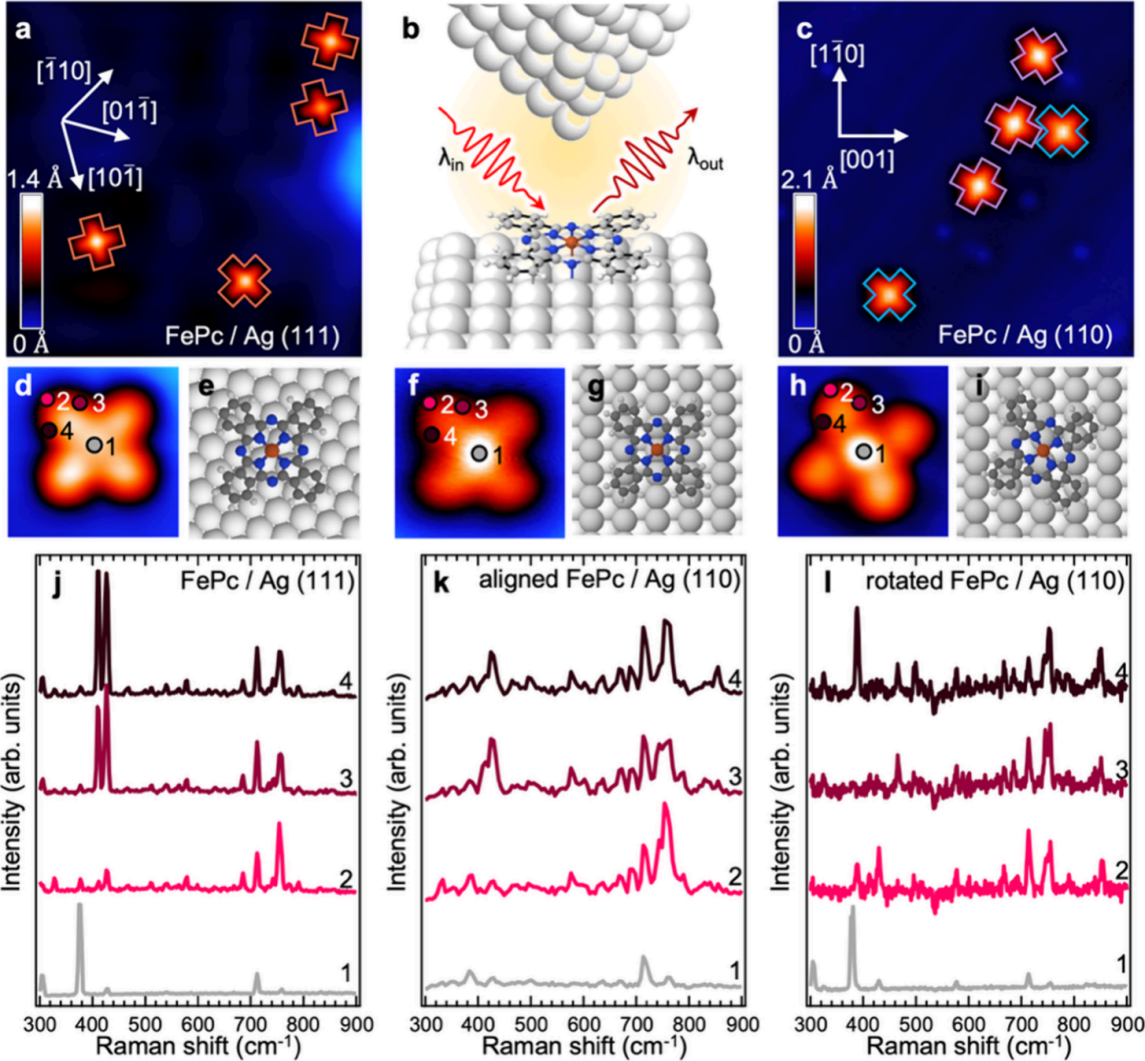
STM topography imaging of the FePc adsorption configurations on Ag surfaces and TERS point spectra. (a) STM image of FePc adsorbed on Ag(111). The main crystallographic directions of the surface are marked with white arrows. The molecules in three equivalent adsorption geometries are marked with orange crosses. Size 15 × 15 nm^2^, set point 50 mV, 30 pA. The scalebar indicates topographic height from 0 to 1.4 Å. (b) Experimental scheme: a laser-illuminated plasmonic tip scans above a FePc molecule on Ag(110) inducing Raman scattering of the light on the molecular vibrational modes. (c) STM image of FePc adsorbed on Ag(110). The aligned and rotated adsorption configurations are denoted with blue and purple crosses, respectively. Size 15 × 15 nm^2^, 100 mV, 16 pA. The scalebar indicates topographic height from 0 to 2.1 Å. (d) Detailed STM image of FePc/Ag(111). Colored circles point to the positions where the TERS spectra have been measured. Size 2.0 × 2.0 nm^2^, set point 1 mV, 200 pA. (e) Ball-and-stick scheme of the FePc/Ag(111) adsorption configuration. (f) High-resolution STM image of aligned FePc/Ag(110). Size 2.0 × 2.0 nm^2^, set point 1 mV, 200 pA. (g) Ball-and-stick scheme of the aligned FePc/Ag(110) adsorption configuration. (h) High-resolution STM image of rotated FePc/Ag(110). Size 2.0 × 2.0 nm^2^, set point 1 mV, 1 nA. (i) Ball-and-stick scheme of the rotated FePc/Ag(110) adsorption configuration. (j–l) Comparison of the spectra taken on FePc (FePc/Ag(111) (j), aligned FePc/Ag(110) (k), and rotated FePc/Ag(110) (l)) at different intramolecular locations color-coded in panels (d), (f), and (h), respectively. The spectra are shifted vertically for clarity. Acquisition parameters: 50 s, 1 mV, 200 pA.

## Results and Discussion

Figure 1a shows an STM image of submonolayer FePc adsorbed on Ag(111) with a characteristic four-lobe and bright-center contrast.^25^ FePc molecules are dispersed on the surface with three equivalent orientations, with one of the main molecular axes (defined by N–Fe–N directions) aligned with any of the three crystallographic low-index ⟨11̅0⟩ directions of the substrate. Alternatively, FePc adsorbed on Ag(110) adopts two nonequivalent configurations: either with the N–Fe–N axes at 45° along the main crystallographic directions (molecules marked with blue crosses in Figure 1c), hereafter referred to as “aligned FePc/Ag(110)”, or rotated approximately ±30° with respect to the [11̅0] crystal directions (purple crosses), referred to as “rotated FePc/Ag(110)” hereafter. Due to the symmetry of the molecule–substrate system, the rotated FePc/Ag(110) is found with its chiral equivalent on the surface (see Figure S2 in the Supporting Information).

For each adsorption configuration, we measure point TERS spectra of the low-wavenumber fingerprint region at four positions: three positions at one lobe and one in the center (marked by colored points and numbers in Figure 1d,f,h). The spectra are presented in Figures 1j, 1k, and 1l for FePc/Ag(111), aligned FePc/Ag(110), and rotated FePc/Ag(110), respectively. We notice that, for each adsorption geometry, the spectra have comparable vibrational mode frequencies, but the relative Raman intensities of the bands strongly vary depending on the location of the measurement above the molecule. Interestingly, for the rotated FePc/Ag(110) system, the TERS spectra obtained on the two opposite peripheral arms of the molecule (locations 3 and 4 in Figure 1h) show significant variations in the relative intensities of the vibrational modes. In contrast, FePc/Ag(111) and aligned FePc/Ag(110) show nearly identical spectra at these locations. Altogether, these results indicate an adsorption-driven nonhomogeneous spatial distribution of the TERS intensity for individual vibrations within the molecules.

To resolve the submolecular intensity distribution in each FePc configuration, we map the TERS signal as a function of the tip position. Real-space, band-resolved TERS mapping pinpoints individual modes within molecules.^12,26–28^ The tip–sample height is decisive to maximize the signal-to-noise ratio. Heights below 50 pm may result in the formation of a molecular point contact (see Figure S3 in the Supporting Information)^29,30^ drastically enhancing the signal but hindering map acquisition. Thus, to avoid the perturbation of the picocavity, our hyperspectral maps are performed at typical mild tunneling conditions with short integration times per pixel (e.g., Figure 2c: 1 mV, 400 pA, 40 × 40 pixels, 2.4 s). The Raman spectra generated by adding all point spectra for a given map are shown in Figure 2a,d,g. From the intensity peaks, we can identify the representative subset of modes, which are TERS-active for all the three adsorption geometries. We create the spatial maps of these modes by integrating the spectral intensities in specific wavenumber windows in Figure 2c,f,i (the full sets of observed band-resolved TERS maps can be found in Figures S4–S7 in the Supporting Information). Each map shows a distinctive pattern with a high level of intramolecular detail, reaching a resolution of 1.6 Å fwhm for the 374 cm^–1^ mode on Ag(111) (see Figure S8 in the Supporting Information). Furthermore, we also distinguish significant variations of intensity distributions in the maps for the same vibrational bands (identified based on similarity of wavenumbers) depending on the adsorption configurations. Surprisingly, we find in many cases that the characteristic mode patterns have a symmetry lower than the expected D_4h_ of the unperturbed molecules, e.g., the band around 745 cm^–1^ in all three cases. In the following, we link the symmetries of the TERS patterns to the anisotropic effect of the surface in each particular adsorption geometry.

**Figure 2:**
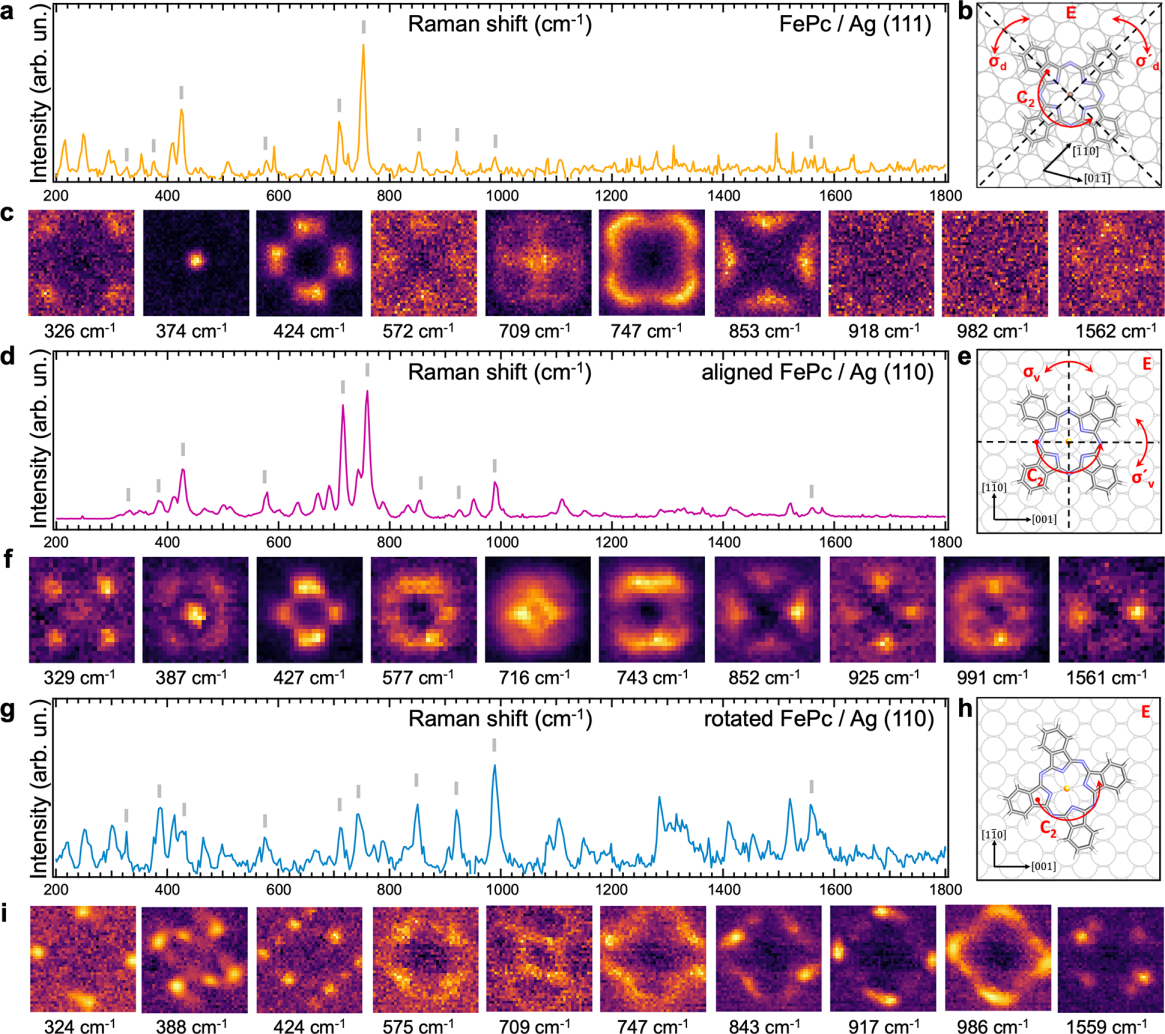
TERS hyperspectral maps of FePc on Ag(111) and (110) terminations. (a) Spatially integrated TERS spectrum of FePc/Ag(111). (b) Scheme of the symmetry elements of the FePc/Ag(111) system: E—identity operator, C_2_—180 ^o^ rotation around the Fe center, and σ_d_ and σ′_d_ mirror planes across the diagonals. (c) Real-space maps of high-intensity vibrations of the FePc/Ag(111). (d) Spatially integrated TERS spectrum of aligned FePc/Ag(110). (e) Scheme of the symmetry elements of the aligned FePc/Ag(110) system: E—identity operator, C_2_—180° rotation around the Fe center, and σ_v_ and σ′_v_ mirror planes across the vertical and horizontal directions, respectively. (f) Real-space maps of high-intensity vibrations of aligned FePc/Ag(110). (g) Spatially integrated TERS spectrum of rotated FePc/Ag(110). (h) Scheme of the symmetry elements of the rotated FePc/Ag(110) configuration: E—identity operator and C_2_—180° rotation around the Fe center. (i) Real-space maps of high-intensity vibrations of rotated FePc/Ag(110).

Upon a careful inspection of the FePc/Ag(111) TERS maps in Figure 2c, we see indications of breaking the D_4h_ rotational symmetry for the 424, 747, and 853 cm^–1^ modes. The apparent symmetry in this case is defined by two mirror planes along the N–Fe–N axes (σ_d_ and σ′_d_ in the simplified model in Figure 2b) and a 2-fold rotation (C_2_ in Figure 2b), indicating an overall C_2d_ point symmetry. The σ_d_ and σ′_d_ mirror axes are aligned with the [11̅0] and [2̅11] surface directions, respectively, coincident with the two types of mirror planes of the top Ag(111) atomic layer and the main anisotropic directions. For the aligned FePc/Ag(110), the picture is different; the majority of the patterns shown in Figure 2f also manifest a 2-fold symmetry with two mirror planes (σ_v_ and σ′_v_ in Figure 2e) now coinciding with the [11̅0] and [001] main crystallographic directions of the Ag(110), indicating a C_2v_ symmetry. Finally, many of the presented TERS maps of the rotated FePc/Ag(110) in Figure 2i clearly have a reduced C_2_ 2-fold rotational symmetry, in agreement with the adsorption model in Figure 2h in which the molecular axes are not aligned with any of the principal crystallographic directions of the surface.

FePc has *N* = 57 atoms and therefore 3*N* – 6 = 165 distinct vibrational modes. In the gas phase, it is represented by the D_4h_ group irreducible representation with Γ = 14A_1g_ + 13A_2g_ + 6A_1u_ + 8A_2u_ + 14B_1g_ + 14B_2g_ + 7B_1u_ + 7B_2u_ + 26E_g_ + 56E_u_. The modes A_1g_, B_1g_, B_2g_, and E_g_ are modes where the molecular vibrations induce a change in the polarizability and therefore are, in principle, Raman-active modes. When lowering the symmetry to C_2d_ and C_2v_, the vibrational modes fall into four irreducible representations A_1_, A_2_, B_1_, and B_2_; upon further reduction to *C*_*2*_ symmetry, the only two irreducible representations left are A and B modes; all of which can be, in principle, Raman active.^2^ Thus, we expect that the anisotropic relaxation of the molecule in the reduced-symmetry adsorption geometries will result in the appearance of new Raman-active modes and the splitting of *D*_4h_ Raman-active degenerate modes (E_g_ modes) in the TERS spectra. We have examined the splitting of the bands in our experiments and found characteristic examples of high-intensity split vibrational bands for the three adsorption configurations (shown in Figure 3).

**Figure 3:**
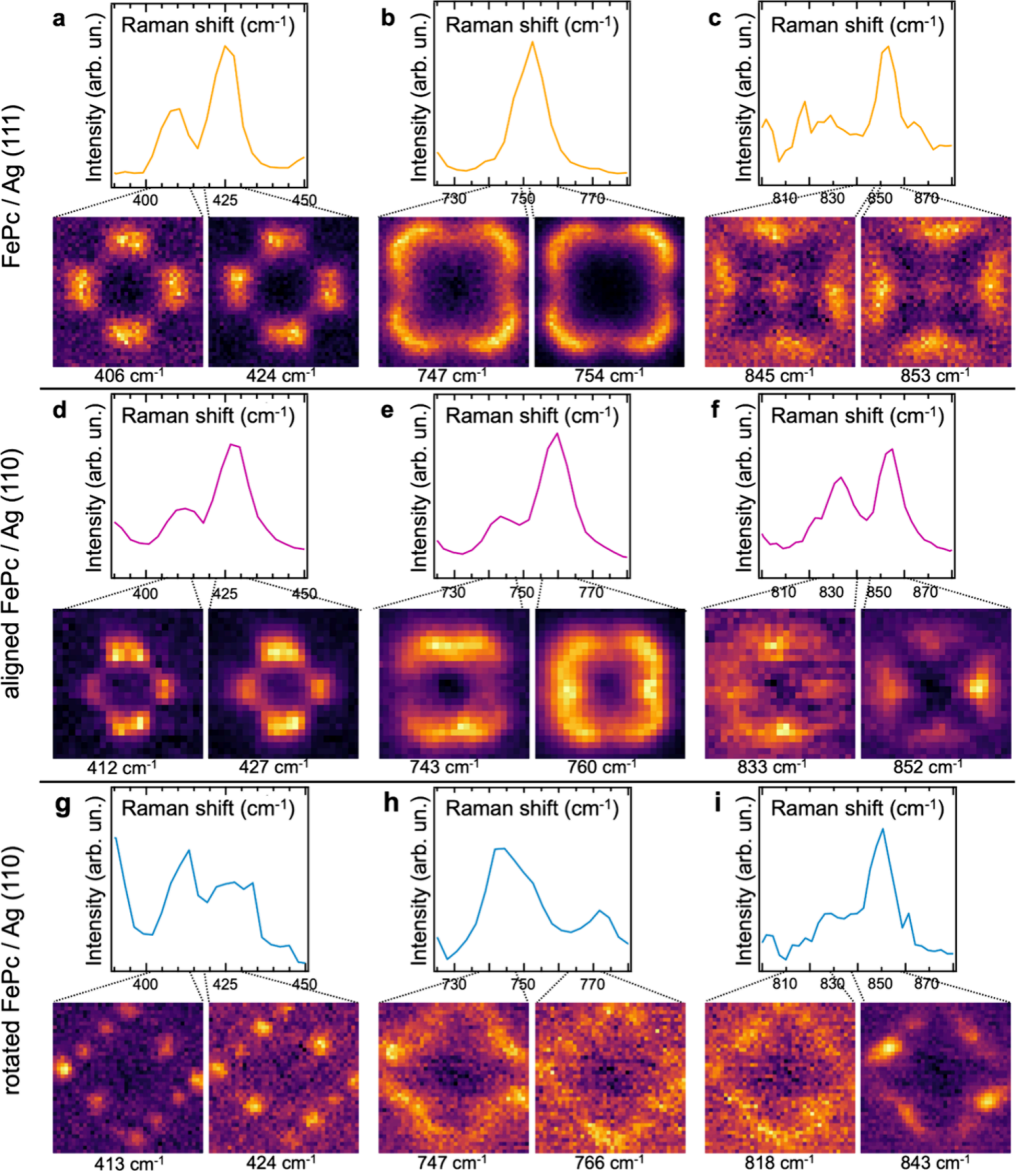
Substrate-induced symmetry lowering and splitting of vibrational modes. (a) Experimentally observed TERS doublet in the 390–450 cm^–1^ region of the FePc/Ag(111) system. The maps corresponding to the Raman intensity of each of the two subpeaks are presented in the lower part. (b) TERS peaks in the 725–780 cm^–1^ region of FePc/Ag(111) and intensity maps of the high- and low-frequency regions of the peak. (c) TERS peaks in the 800–880 cm^–1^ region of the FePc/Ag(111) and intensity maps. (d) TERS doublet in the 390–450 cm^–1^ region of the aligned FePc/Ag(110) configuration and intensity maps. (e) TERS doublet in the 725–780 cm^–1^ region of the aligned FePc/Ag(110) configuration and intensity maps. (f) TERS doublet in the 800–880 cm^–1^ region of the aligned FePc/Ag(110) configuration and intensity maps. (g) TERS doublet in the 390–450 cm^–1^ region of the rotated FePc/Ag(110) configuration and intensity maps. (h) TERS doublet in the 725–780 cm^–1^ region of the rotated FePc/Ag(110) configuration and intensity maps. (i) TERS doublet in the 800–880 cm^–1^ region of the rotated FePc/Ag(110) configuration and intensity maps.

In Figure 3a,d,g, we present vibrations in the range 380–450 cm^–1^ for the three adsorption configurations, which we attribute to two D_4h_ intrinsically degenerate out-of-plane displacements of the atoms belonging to the benzene units (the two degenerate E_g_ modes at 419 cm^–1^ shown in Figure S9 in the Supporting Information), according to our gas-phase DFT calculations. The second column (Figure 3b,e,h) presents the vibrational band 720–780 cm^–1^ corresponding to E_g_ modes at 764 cm^–1^ in Figure S9. The third column (Figure 3c,f,i) shows the region 800–880 cm^–1^ related to out-of-plane deformation of benzene rings (E_g_ modes at 872 cm^–1^ in Figure S9). For FePc in C_2d_ and C_2v_ configurations, we are able to resolve two distinct TERS maps with an orthogonal contrast along one of the two mirror planes within a few tens of wavenumbers. This is a clear indication that these doublets come from vibrational modes whose original degeneracy has been lifted.^5^ The value of the frequency split of each pair of degenerate vibrations is proportional to the magnitude of the anisotropy of the local potential surface and correlates with the amplitude of the adsorption-induced structural deformation and the anisotropic redistribution of charge and hybridization with the substrate, which modifies the Raman polarizability. We also observe minor shifts of few cm^–1^ for the modes from one adsorption configuration to another, which can be related to vibrational softening/stiffening. For molecules in the C_2_ configuration, the stronger symmetry reduction produces complex chiral patterns in the hyperspectral maps of the split modes and weaker Raman intensities. Overall, we attribute the band splitting and shifts of the originally degenerated Raman-active E_g_ modes to the atomic distortion and concomitant molecular orbital hybridization occurring in response to the interaction with the metal substrate. In Figure S10, we introduce the theoretical atomic displacements of the calculated frequencies presented in Figure 3. This notion is further supported by the comparison with TERS spectra measured on FePc molecules adsorbed on two monolayers of sodium chloride (NaCl), maintaining a D_4h_ symmetry, where vibrational splitting (degeneracy lifting) is not observed (see Figure S11 in the Supporting Information).

To confirm our interpretation, we carried out DFT calculations of the different adsorption geometries of FePc on the Ag(111) and Ag(110) surfaces. The lowest-energy calculated configurations reproduce the experimentally observed adsorption configurations (see Figures S12 and S13 in the Supporting Information) in agreement with previous experimental results and calculations.^31,32^ In Figure 4, we introduce the schematics of the atomic positions for each configuration, with the side views being aligned with the high-density symmetry directions of the surface. The individual atoms are color-coded according to their out-of-plane height coordinate. FePc/Ag(111) adopts a bowl-shaped configuration having the center Fe atom 71 pm lower than the highest H atom of the benzene extrema. Aligned FePc/Ag(110) adopts a saddle-shape geometry where two N atoms are the closest to the surface and H atoms on the orthogonal benzene rings lying 59 pm higher. At last, rotated FePc/Ag­(110) adopts a configuration where all four benzene rings are distorted to form a propeller-like shape, with the difference between the lowest and highest atoms being 68 pm. Calculations thus confirm that the molecules break their original D_4h_ symmetry and relax to a lower-symmetry configuration due to the interaction with the surface. The analysis of the symmetry of the optimized structures confirms the new molecular point groups (i.e., C_2d_, C_2v_, and C_2_). We also note that strong charge transfers from the surface to the molecule of ca. 0.8 e^–^ are predicted, indicating that FePc tends to spontaneously charge on the surface. While the total donated charge is similar, the spatial distribution of that charge is markedly different (see Figure S14 in the Supporting Information). The charge distribution in FePc/Ag(111) is primarily centered around the Fe metal atom, first neighboring N atoms, and second-neighbor carbon atoms, leaving the four benzene extrema electronically unperturbed. On the contrary, for the two configurations of FePc on Ag(110), the charge redistribution is spread out across the whole molecule, showing both the metal center and the aromatic macrocycle (including the outermost carbon rings), regions of increased/decreased electron density. This relatively strong charge transfer amplifies the symmetry breaking through charge redistribution effects already present due to adsorption, leading to additional splitting of E_g_ modes and mode-specific frequency shifts, which may further explain the observed discrepancies between our submolecularly resolved TERS spectra and the Raman spectra obtained previously on FePc thin films.^33,34^

**Figure 4:**
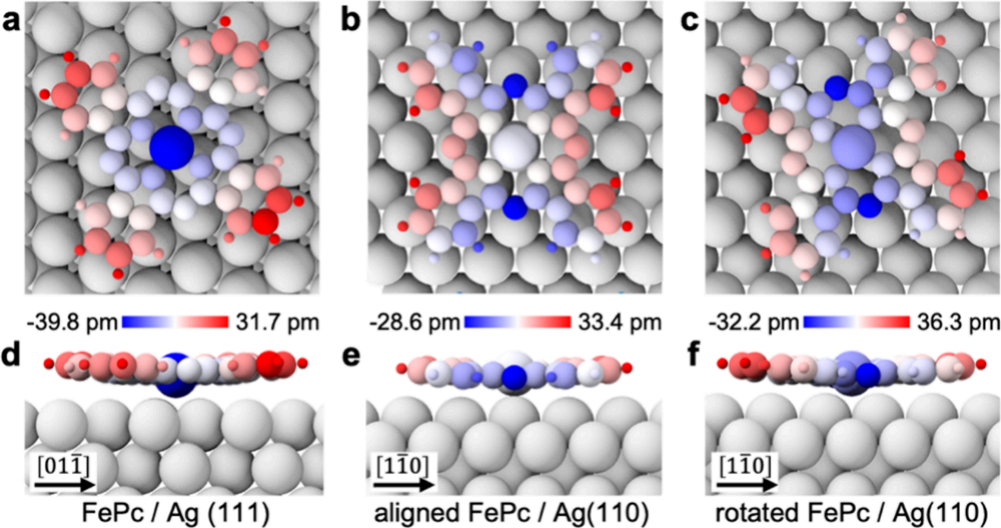
Adsorption-induced out-of-plane relaxations and symmetry breaking calculations obtained with density functional theory. (a–c) Front views of the optimized geometries for the FePc/Ag(111) (a), aligned FePc/Ag(110) (b), and rotated FePc/Ag(110) (c) configurations. (d–f) Side views of the optimized FePc/Ag(111) (d), aligned FePc/Ag(110) (e), and rotated FePc/Ag(110) (f) geometries. The height (*z*) atomic displacements are color-coded with respect to the average *z*-coordinate of the molecule: atoms located below the mean height are shown in blue, while those above it are shown in red (closer to or further from the Ag surface).

Our results have implications for the nascent field of chemical recognition of molecules with submolecularly resolved hyperspectral TERS maps. We demonstrate that it is possible to detect the adsorption-induced symmetry breaking and discover subångstrom structural displacements by experimental optical means.^35^ We anticipate that this phenomenon will also be observable for molecules undergoing Jahn–Teller distortions.^36^ We also show that the effect of the substrate and the resulting overall chirality can severely alter the expected optical properties at the picoscale,^37–39^ and that the symmetry of the substrate must be considered when reconstructing the chemical structure of an unknown adsorbate from TERS measurements. We anticipate future theoretical work on the interpretation and simulation of the substrate-induced distortions of Ångstrom-resolved Raman maps.^40^

## Conclusions

In conclusion, we have characterized the vibrational modes of individual FePc molecules adsorbed on two low-index Ag crystal surface terminations by means of TERS with a submolecular resolution. The vibrational mode patterns were successfully analyzed for three adsorption configurations that represent a gradual reduction of the original D_4h_ symmetry of the molecule to C_2d_, C_2v_, and C_2_ symmetries, depending on the surface termination and the molecular in-plane rotational angle on it. We have demonstrated that symmetry breaking has a strong impact on the observed hyperspectral patterns, proving that TERS is sensitive to picometer-scale distortions of the positions of the atoms of the adsorbates. We have identified the vibrational modes for which the degeneracy has been lifted, revealing how subtle variations in the adsorption geometry and registry with the underlying substrate profoundly affect the activity of the molecular vibrations, which is relevant to control on-surface reactions with enhanced chemoselectivity. Our work creates a basis for precisely studying point-group symmetries of single molecules by using purely optical methodologies.

## Supplementary Material



## Notes

Methods, experimental details, extended microspectroscopic data, and extended calculations (PDF)

## References

[1] Larkin, P. Infrared and Raman Spectroscopy: Principles and Spectral Interpretation. 2nd Ed.; Elsevier: Amsterdam, Netherlands, 2017. 10.1016/C2015-0-00806-1

[2] Sforzini J., Bocquet F. C., Tautz F. S. (2017). Adsorption-induced symmetry reduction
of metal-phthalocyanines studied by vibrational spectroscopy. Phys. Rev. B.

[3] Uhlmann C., Swart I., Repp J. (2013). Controlling the Orbital
Sequence
in Individual Cu-Phthalocyanine Molecules. Nano
Lett..

[4] Moskovits M. (1982). Surface selection
rules. J. Chem. Phys..

[5] Chiang N. (2017). Probing Intermolecular
Vibrational Symmetry Breaking in Self-Assembled
Monolayers with Ultrahigh Vacuum Tip-Enhanced Raman Spectroscopy. J. Am. Chem. Soc..

[6] Ohta N., Arafune R., Tsukahara N., Takagi N., Kawai M. (2014). Adsorbed states
of iron­(II) phthalocyanine on Ag(111) studied by high-resolution electron
energy loss spectroscopy. Surf. Interface Anal..

[7] Kumagai T., Miwa K., Cirera B. (2025). Point-Contact
Tip-Enhanced Raman
Spectroscopy: Picoscale Light–Matter Interactions within Plasmonic
Cavities. Nano Lett..

[8] Pozzi E. A. (2017). Ultrahigh-Vacuum Tip-Enhanced
Raman Spectroscopy. Chem. Rev..

[9] Zrimsek A. B. (2017). Single-Molecule Chemistry
with Surface- and Tip-Enhanced Raman Spectroscopy. Chem. Rev..

[10] Itoh T. (2023). Toward a New Era of SERS and TERS at the Nanometer
Scale: From Fundamentals
to Innovative Applications. Chem. Rev..

[11] Li L. (2022). Chemically identifying
single adatoms with single-bond sensitivity
during oxidation reactions of borophene. Nat.
Commun..

[12] Lee J., Crampton K. T., Tallarida N., Apkarian V. A. (2019). Visualizing vibrational
normal modes of a single molecule with atomically confined light. Nature.

[13] Jaculbia R. B. (2020). Single-molecule resonance Raman effect in a
plasmonic nanocavity. Nat. Nanotechnol..

[14] Cirera B. (2022). Charge Transfer-Mediated
Dramatic Enhancement of Raman Scattering
upon Molecular Point Contact Formation. Nano
Lett..

[15] Wang R.-P. (2021). Raman Detection of Bond Breaking and Making of a Chemisorbed Up-Standing
Single Molecule at Single-Bond Level. J. Phys.
Chem. Lett..

[16] Chen Z. (2019). Operando Characterization of Iron Phthalocyanine Deactivation during
Oxygen Reduction Reaction Using Electrochemical Tip-Enhanced Raman
Spectroscopy. J. Am. Chem. Soc..

[17] Han Y. (2024). Real-Space Spectral
Determination of Short Single-Stranded DNA Sequence
Structures. J. Am. Chem. Soc..

[18] Zhu L.-Y. (2025). Algorithm-Assisted Structure
Identification of Individual Nucleobases
in Single DNA Chains through Tip-Enhanced Raman Spectromicroscopy. JACS Au.

[19] de
Campos Ferreira R. C. (2024). Resonant Tip-Enhanced Raman Spectroscopy
of a Single-Molecule Kondo System. ACS Nano.

[20] Luo Y. (2023). Imaging and controlling
coherent phonon wave packets in single graphene
nanoribbons. Nat. Commun..

[21] Cuadrado R. (2010). CoPc adsorption on Cu(111): Origin of the C4
to C2 symmetry reduction. J. Chem. Phys..

[22] Chang S.-H. (2008). Symmetry reduction of
metal phthalocyanines on metals. Phys. Rev.
B.

[23] Scheuerer P. (2019). Charge-Induced Structural
Changes in a Single Molecule Investigated
by Atomic Force Microscopy. Phys. Rev. Lett..

[24] Frankerl M. (2025). Substrate Polarization
Alters the Jahn-Teller Effect in a Single
Molecule. Phys. Rev. Lett..

[25] Mugarza A. (2012). Electronic and magnetic
properties of molecule-metal interfaces:
Transition-metal phthalocyanines adsorbed on Ag(100). Phys. Rev. B.

[26] Xu J. (2021). Determining structural and chemical heterogeneities
of surface species
at the single-bond limit. Science.

[27] Zhang R. (2013). Chemical mapping of
a single molecule by plasmon-enhanced Raman scattering. Nature.

[28] Zhang Y. (2019). Visually constructing the chemical structure of a single molecule
by scanning Raman picoscopy. Natl. Sci. Rev..

[29] Cirera B., Wolf M., Kumagai T. (2022). Joule Heating
in Single-Molecule
Point Contacts Studied by Tip-Enhanced Raman Spectroscopy. ACS Nano.

[30] Liu S. (2020). Dramatic Enhancement of Tip-Enhanced Raman Scattering Mediated by
Atomic Point Contact Formation. Nano Lett..

[31] Sedona F. (2012). Tuning the catalytic
activity of Ag(110)-supported Fe phthalocyanine
in the oxygen reduction reaction. Nat. Mater..

[32] Bartolomé E. (2020). Enhanced Magnetism through
Oxygenation of FePc/Ag(110) Monolayer
Phases. J. Phys. Chem. C.

[33] Corio P., Rubim J. C., Aroca R. (1998). Contribution
of the Herzberg–Teller
Mechanism to the Surface-Enhanced Raman Scattering of Iron Phthalocyanine
Adsorbed on a Silver Electrode. Langmuir.

[34] Kumar A. (2018). Influence of substrate
on molecular order for self-assembled adlayers
of CoPc and FePc. J. Raman Spectrosc..

[35] Lee J., Tallarida N., Chen X., Jensen L., Apkarian V. A. (2018). Microscopy
with a single-molecule scanning electrometer. Sci. Adv..

[36] Yu H.-Z. (2026). Theoretical prediction for monitoring Jahn-Teller vibrational evolution
using real-space tip-enhanced Raman imaging. Nat. Commun..

[37] Fiederling K. (2023). A Full Quantum Mechanical
Approach Assessing the Chemical and Electromagnetic
Effect in TERS. ACS Nano.

[38] Tang Z.-X., Zhang Y., Dong Z.-C. (2025). Theoretical
study of tip-enhanced
Raman optical activity for a single chiral molecule. Opt. Commun..

[39] Chulhai D. V., Jensen L. (2013). Determining Molecular Orientation
with surface-enhanced
Raman scattering using inhomogenous electric fields. J. Phys. Chem. C.

[40] Chen X., Liu P., Hu Z., Jensen L. (2019). High-resolution tip-enhanced Raman
scattering probes sub-molecular density changes. Nat. Commun..

