# Supplementary material for: Adsorption-Driven Symmetry Lowering in Single Molecules Revealed by Ångstrom-Scale Tip-Enhanced Raman Imaging
Source: J Am Chem Soc. 2026 Feb 27;148(9):9375–81. doi: 10.1021/jacs.5c18593 (PMC12983298; doi:10.1021/jacs.5c18593)
Supplement: Supplementary file 1 [file ja5c18593_si_001.pdf]

Supporting Information for

Adsorption-Driven Symmetry Lowering in Single Molecules Revealed by Ångstrom-scale Tip-Enhanced Raman Imaging

Rodrigo Cezar de Campos Ferreira[1,2 ǂ], Borja Cirera[3 ǂ], Jiří Doležal[1], Álvaro Gallego de Roa[4], Amandeep Sagwal[2,5], Petr Kahan[2], Rubén Canales[3], Fernando Aguilar-Galindo[4,6]*, Martin Švec[1,2]*, and Pablo Merino[3]*

[1] Institute of Organic Chemistry and Biochemistry, Czech Academy of Sciences, Praha 6 CZ16000, Czech Republic

[2] Institute of Physics, Czech Academy of Sciences, Praha 6 CZ16200, Czech Republic

[3] Instituto de Ciencia de Materiales de Madrid (ICMM-CSIC), Madrid ES28049, Spain

[4] Departamento de Química, Universidad Autónoma de Madrid, Madrid ES28049,Spain

[5] Faculty of Mathematics and Physics, Charles University; Ke Karlovu 3, CZ12116 Praha 2, Czech Republic

[6] Institute for Advanced Research in Chemical Sciences (IAdChem), Universidad Autónoma de Madrid, Madrid ES28049, Spain

ǂ authors contributed equally

Email: fernando.aguilar-galindo@uam.es

Email: svec@fzu.cz

Email: pablo.merino@csic.es

Table of contents

1. Methods
2. Scheme of the confocal optical setup for TERS measurements
3. Adsorption configurations on Ag(110)
4. TERS evolution upon molecular point contact
5. Additional Raman Maps for the three different configurations
6. Raman Maps of additional split modes.
7. Lateral spatial resolution of the TERS Mapping
8. Vibrational analysis of Gas-Phase FePc
9. Vibrational analysis of FePc anions at selected frequencies.
10. TERS measurements on FePc/NaCl/FePc(111)
11. Density Functional Theory calculations of minimum energy configurations
12. Density Functional Theory Analysis of Charge Transfer in FePc/Ag Interfaces
13. Supporting references

1. Methods.

Tip-enhanced Raman Spectrometry in ultrahigh vacuum and low temperatures: All experiments were carried out in a low-temperature scanning tunneling microscope (LT-STM, Createc GmbH) operated at 7 K under ultrahigh vacuum (UHV) conditions with a base pressure below 5×10^{-11}mbar. The Ag(111) substrate was prepared by repeated cycles of Ar^+ sputtering and annealing at 550 °C. Iron phthalocyanine (FePc) molecules were thermally sublimated at 390 °C onto Ag(111) and Ag(110) surfaces held at 5 K.The optical excitation was provided by a continuous-wave He–Ne laser (λ_{in}= 632.8 nm). The beam was collimated with a 15 mm focal length lens and passed through a neutral density filter, half-wave plate, polarizer, and noise eater to ensure stable intensity and polarization along the tip–sample axis. The laser power at the sample was 50–200 µW. Focusing into the STM junction was achieved with an internal 15 mm lens. Emission from the plasmonic picocavity was collected and filtered with a 633 nm bandpass edge filter. Tip-enhanced Raman scattering (TERS) spectra were acquired in cumulative mode using an Andor Kymera 328i spectrograph (1200 grooves/mm grating, 500 nm blaze) controlled by custom software built on the Andor SDK (see Ref. [41] for details). Ag tips were fabricated from 25 µm diameter wire, sharpened by focused Xe^+ ion beam milling, and subsequently cleaned by head-on Ar^+ sputtering. Final tip shaping was achieved in situ via nanoindentations and voltage pulses. The picocavity resonance spectrum was characterized by electroluminescence and tuned to overlap with the excitation energy and Raman scattering range. Efficient photon coupling to the picocavity was verified through field-emission resonance measurements using a lock-in technique. Coupling efficiency was inferred from shifts in electron tunneling resonances that coincided with the excitation energy.[42]

The hyperspectral TERS maps were performed at constant current - feedback system turned on- using setpoints from 2.5 pA to 2 nA and few mV of bias voltage which optimized the signal-to-noise ratio for the given experimental picocavity. After selecting a single molecule of a particular orientation, a grid of 25 x 25 points (or 40 x 40 points) of individual point spectra were taken using the defined setpoint. Each spectrum in the hyperspectral map was integrated during 2.4 to 10 s. Once the hyperspectral data cube is obtained, we perform frequency slicing to obtain the Raman maps presented in the manuscript using a home-made code permitting to represent the intensity of the Raman signal for a given frequency and real-space pixel. The selected maps are optimized by selecting the central frequency (the number displayed below the Raman maps) and the frequency integration window (3 to 5 cm^{-1}).

Density Functional Theory Methods:

Theoretical calculations were performed within the framework of Density Functional Theory (DFT) using two complementary dispersion-inclusive approaches, chosen according to the physical nature of the systems studied and the capabilities of the respective computational codes.

Periodic calculations for the adsorbed FePc/Ag systems were carried out using the Vienna Ab Initio Simulation Package (VASP) [43]. The optPBE exchange functional [44] within the van der Waals density functional (vdW-DF)[45] framework was employed, which incorporates nonlocal dispersion interactions self-consistently and is well established for describing molecule-surface adsorption geometries. The electronic wavefunctions were expanded in a plane-wave basis set up to a kinetic energy cutoff of 420 eV, and electron-ion interactions were described using projector-augmented wave (PAW) pseudopotentials, as provided by the VASP database. The electronic self-consistency cycle was converged to 10-5 eV. Atomic positions of the molecule and z-coordinates of the first Ag layer were relaxed until all Hellmann-Feynman forces were below 0.01 eV Å-1. The Ag substrates were modeled as slabs consisting of four atomic layers

arranged in 6×8 and 5×7 supercells for the Ag(110) and Ag(111) surfaces, respectively. A vacuum region of approximately 15 Å was introduced along the surface normal to avoid interactions between periodic images. Brillouin zone sampling was restricted to the Γ-point.

Vibrational calculations for the isolated FePc molecule in the gas phase (D_{4h} symmetry) were performed using Gaussian 16. Geometry optimizations and vibrational frequency calculations were carried out using the PBE functional [46] augmented with Grimme's D3 dispersion correction [47] including Becke-Johnson damping (PBE-D3BJ),[48] in order to retain a consistent GGA+dispersion description for the molecular system, since the optPBE functional is not implemented in Gaussian. The cc-pVDZ basis set [49] was employed for the gas-phase calculations.

2. Scheme of the confocal optical setup for TERS measurements.

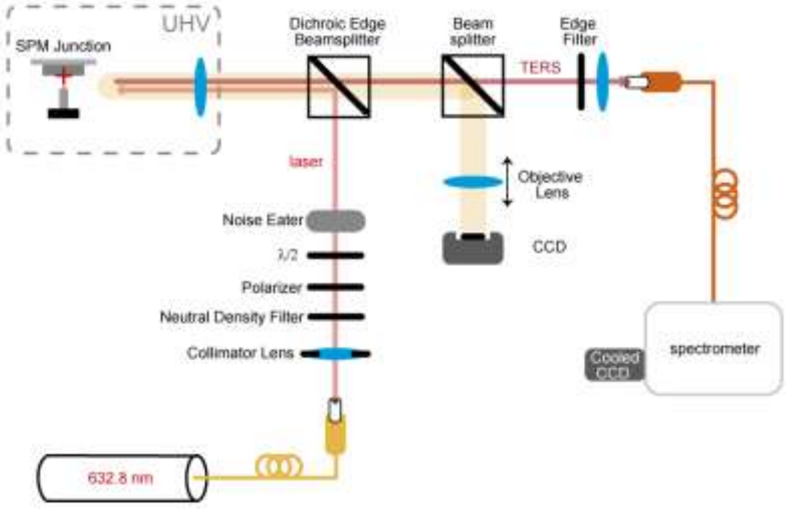

Figure S1. *Scheme of the confocal optical setup for TERS measurements in a ultrahigh vacuum cryogenic scanning probe microscope used in our experiments. The home-built setup has three subsystems: the laser source focusing on the tip-sample junction, a CCD camera for coarse adjustment and a collection system collimating the scattered signal to a fiber which is coupled to a spectrometer.*

3. Adsorption configurations on Ag(110) and Ag(111).

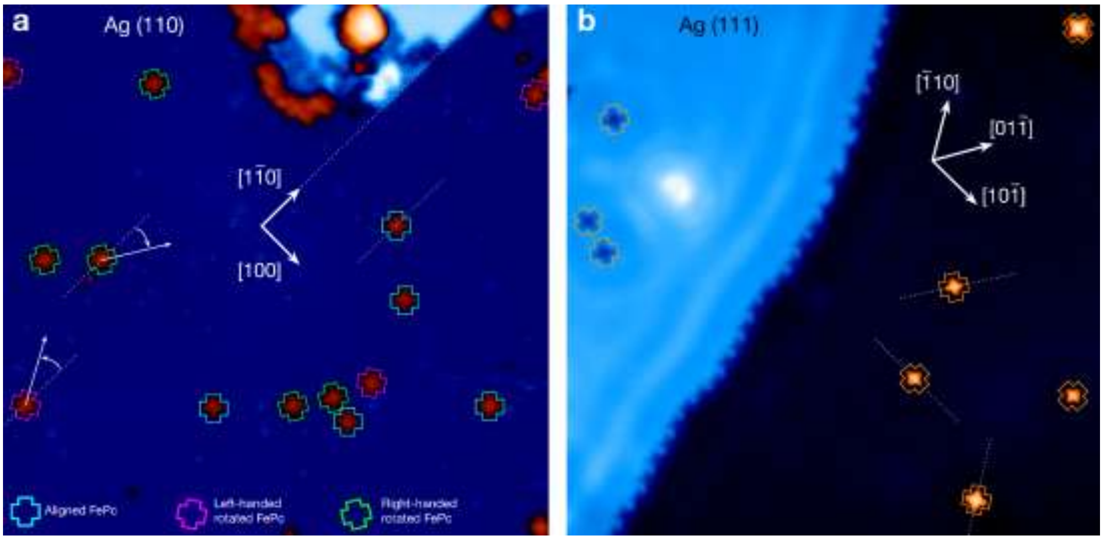

Figure S2. *Overview STM images showing the main adsorption configurations of FePc on Ag(110) and Ag(111). a) The two equivalent surface induced chirality orientations of the rotated FePc/Ag(110) are highlighted by green and magenta crosses for right-handed and left-handed adsorbates respectively. The aligned FePc/Ag(110) molecules are highlighted in cyan crosses for comparison. 40 x 40 nm* ^{2}*, setpoint 10 mV, 10 pA. b) All equivalent orientations of FePc adsorbed on Ag(111). 40 x 40 nm*^{2}*, setpoint 50mV, 27 pA.*

4. TERS evolution upon molecular point contact.

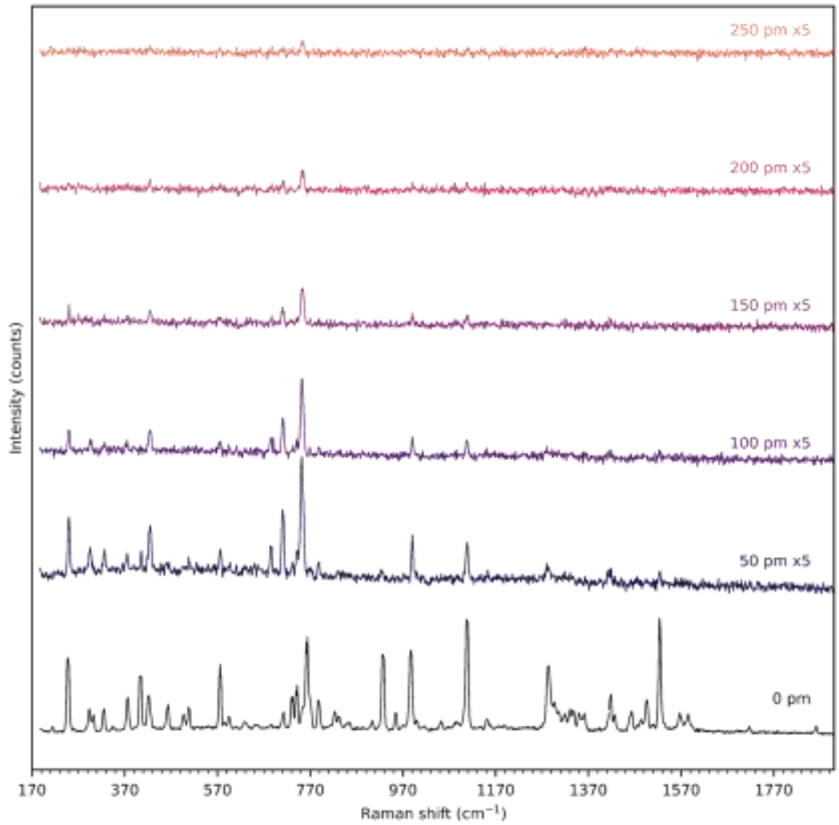

Figure S3. *Height-dependent TERS spectra obtained approaching the tip on the lobe of a rotated FePc/Ag(110) adsorbate (λ_{in} = 632.8 nm, 10 mV). The relative tip-height above the sample with respect to the contact point is specified in picometers (pm) on the right-hand side of every spectrum together with the magnification factor of the spectra (5x). The strong enhancement of the signal (see the black curve at the bottom of the plot with reference tip height of 0 pm) occurs at the onset of the point-contact regime. Our experimental maps and point spectra were performed in the tunneling regime typically at distances of tens of pm above point contact.*

5. Additional Raman maps for the three different configurations.

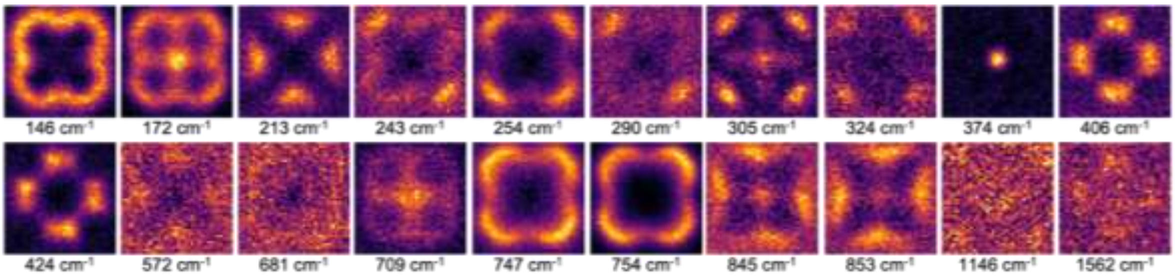

Figure S4. *Wavenumber-resolved slices extracted from submolecular-resolution TERS hyperspectral datasets, highlighting the most intense vibrational modes of FePc/Ag(111). Individual spectra were taken on a grid of 40 x 40 points, in an area of 2.0 × 2.0 nm^2, using a constant-current mode with the setpoint of 1 mV, 400 pA. Each spectrum in the hyperspectral map was integrated during 2.4 s. The maps integrate the Raman intensity of the central wavelength, number diplayed below each map, and the signal of a ± 1.5 cm^{-1} window.*

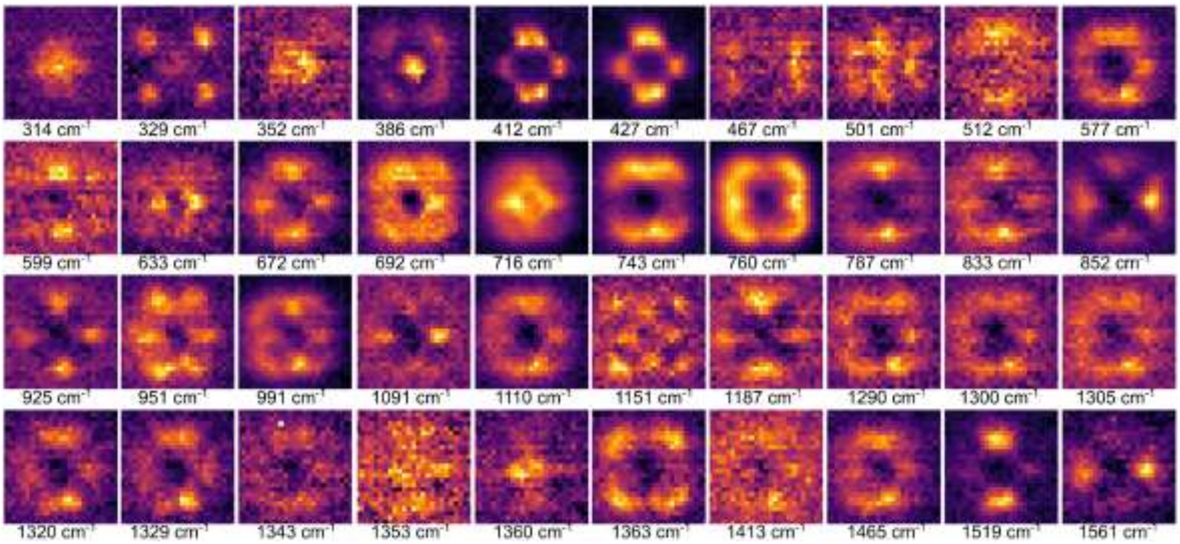

Figure S5. *Wavenumber-resolved slices extracted from submolecular-resolution TERS hyperspectral datasets, highlighting the most intense vibrational modes of aligned FePc/Ag(110). Individual spectra were taken on a grid of 25 x 25 points, in an area of 2.5 × 2.5 nm^2, using a constant-current mode with the setpoint of 10 mV, 2 nA. Each spectrum in the hyperspectral map was integrated during 10 s. The maps integrate the Raman intensity of the central wavelength, number displayed below each map, and the signal of a ± 1.5 cm^{-1} window.*

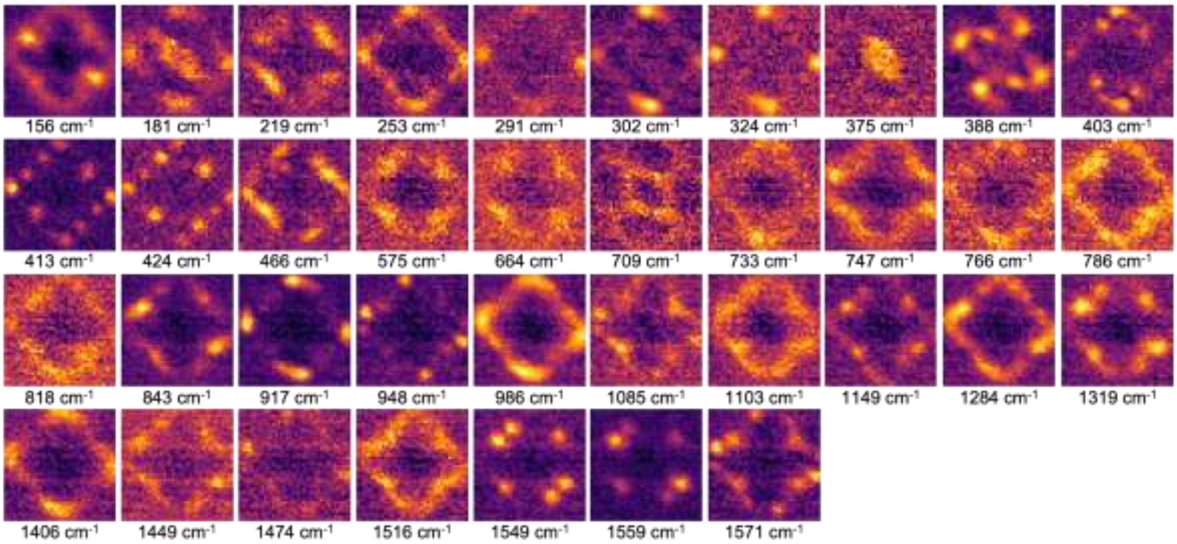

Figure S6. *Wavenumber-resolved slices extracted from submolecular-resolution TERS hyperspectral datasets, highlighting the most intense vibrational modes of rotated FePc/Ag(110). Individual spectra were taken on a grid of 40 x 40 points, in an area of 2.0 × 2.0 nm^2, using a constant-current mode with the setpoint of 10 mV, 2.5 pA. Each spectrum in the hyperspectral map was integrated during 3.5 s. The maps integrate the Raman intensity of the central wavelength, number displayed below each map, and the signal of a ± 1.5 cm^{-1} window.*

6. Raman maps of additional split modes.

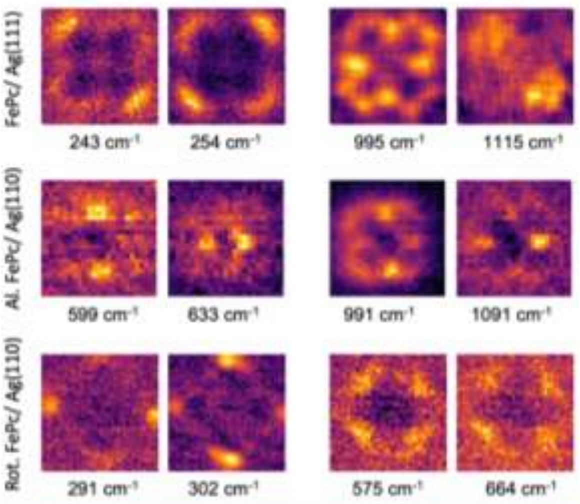

Figure S7. *Substrate-induced symmetry-lowering and splitting of vibrational modes. Experimentally observed TERS doublets for the FePc adsorbed on the Ag(110) and Ag(111) system. The maps corresponding to the Raman modes originating from degenerate E_G modes that split upon adsorption.*

7. Lateral spatial resolution of the TERS Mapping

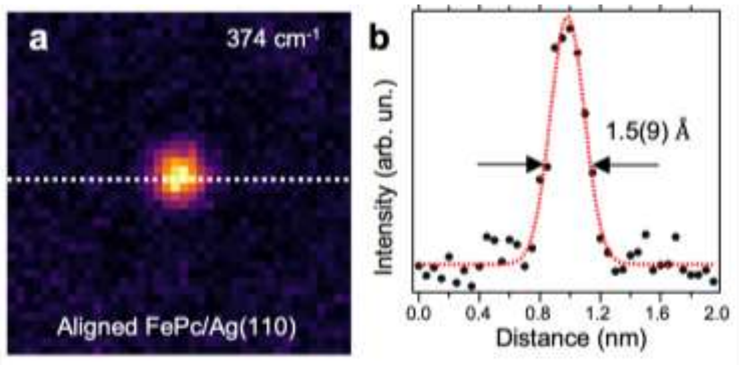

Figure S8. *Determination of TERS spatial resolution in our experiments.* ***a****. Hyperspectral TERS wavenumber slice at 374 cm^{-1} showing a single feature centered on the Fe atom of the FePc molecule.* ***b****. The corresponding TERS intensity profile along the dashed line in panel a) with a fitted Gaussian curve permitting to determine a full width half maximum (FWHM) of 1.59 Å.*

8. Vibrational analysis of gas-phase FePc

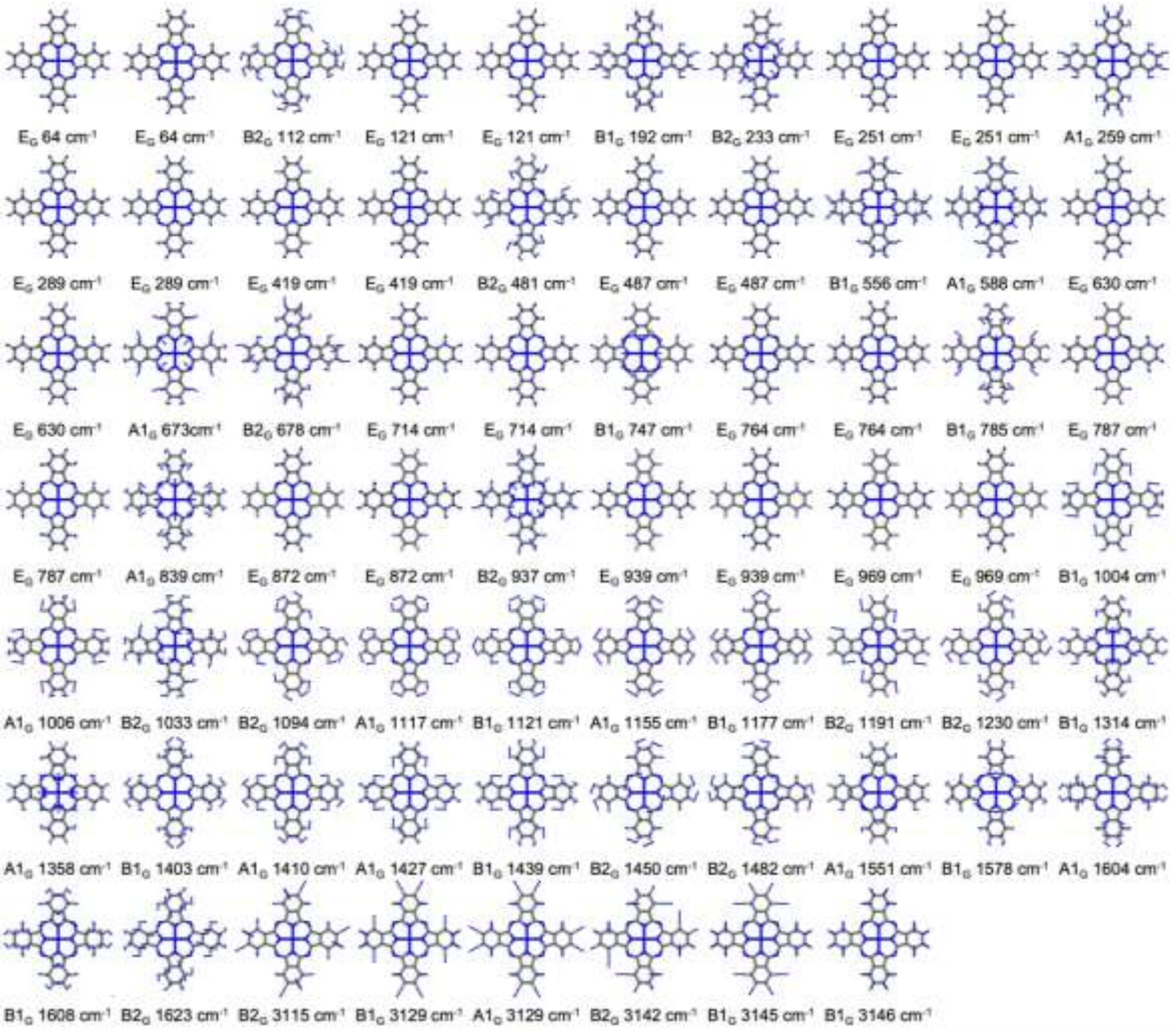

Figure S9. *Raman active normal modes of the FePc in the gas phase. Atomic vibrational displacements are represented by blue arrows for each mode, with the symmetry of the mode and the corresponding wavenumber indicated below. The irreducible representations contributing to Raman activity are 14* A_{1G} *, 14*B_{1G} *, 14*B_{2G} *and 26*E_G*, accounting for a total of 68 modes. We note that* E_G *modes appear in degenerate doublets. The calculated vibrational energy scale has not been corrected* [50] *and wavenumbers are as extracted from DFT calculations.*

9. Vibrational analysis of FePc anions at selected frequencies.

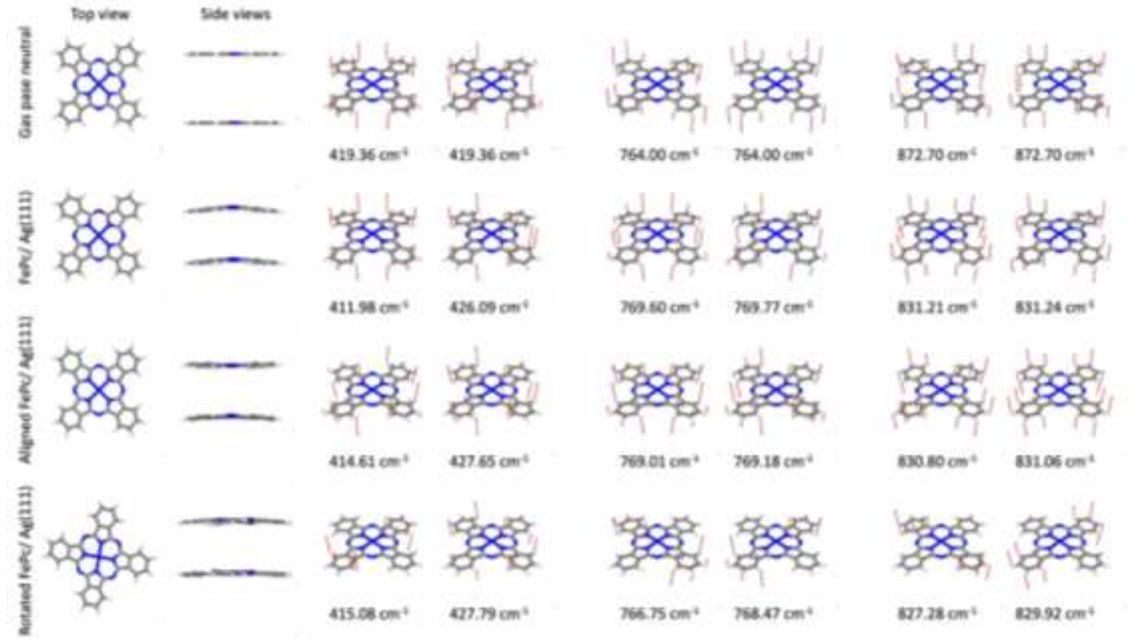

Figure S10. *Atomic displacements of the vibrational modes presented in Fig.3 of the main manuscript. DFT calculations are made using the geometrical distortions attained after relaxation in the three adsorption configurations and in the anionic state (except for the gas phase calculation in the top raw, which is in the neutral state for comparison).*

10. TERS measurements on FePc/NaCl/FePc(111)

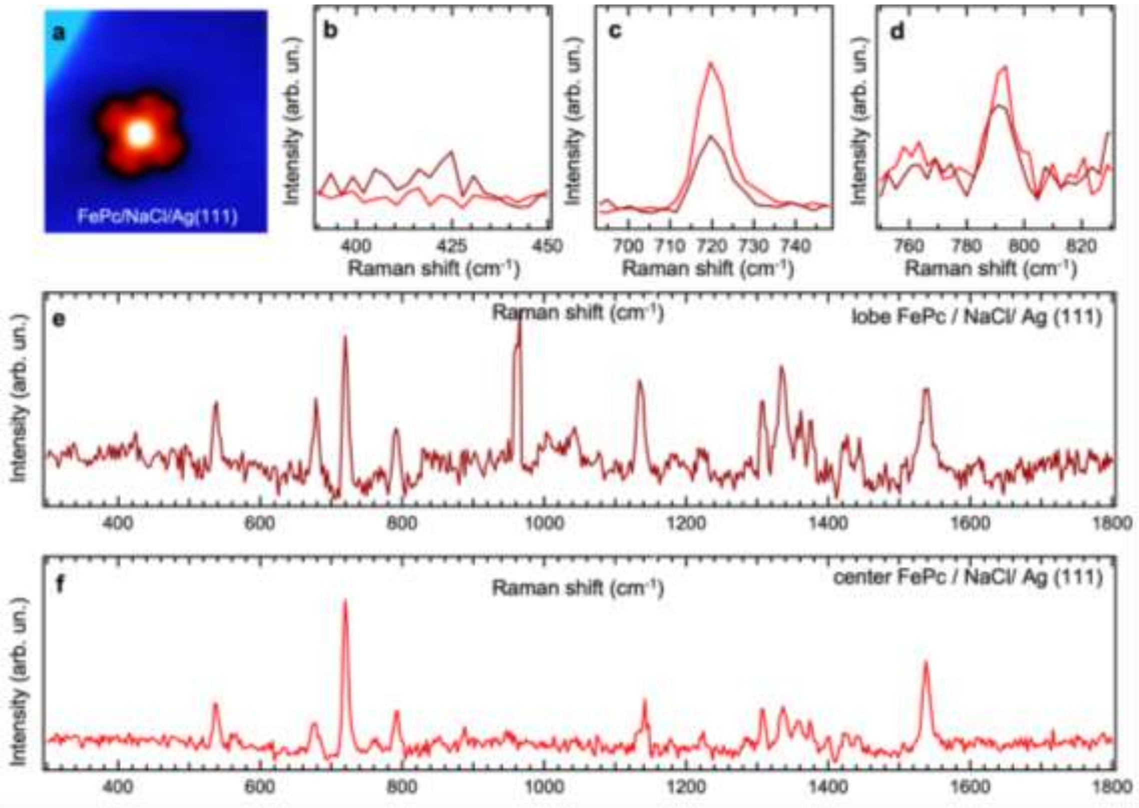

Figure S11. a. *STM image of a single FePc molecule adsorbed on a bilayer of NaCl grown on Ag(111). The molecule is aligned with the main crystallographic directions of the NaCl that can be determined from the terrace step at the upper left corner of the image (2 pA, 10 mV, 5 x 5 nm^2).* ***b***. *Experimentally observed TERS spectra in the 390 cm^{-1} to 450 cm^{-1} region of the FePc/NaCl/Ag(111) system. The two spectra correspond to the TERS intensity measured by placing the tip on the center (red line) and the lobe (brown line).* ***c.*** *TERS peaks in the 698-748 cm^{-1} range on the center and on the lobe of FePc/NaCl/Ag(111) that we assign to the modes shown in the 720-780 cm^{-1} region in Fig. 3 of the main manuscript.* ***d.*** *TERS peaks in the 750-830 cm^{-1} on the center and on the lobe of FePc/NaCl/Ag(111) that we assign to the modes shown in the 800-880 cm^{-1} region in Fig. 3 of the main manuscript.* ***e***. *TERS spectrum of the FePc/NaCl/Ag(111) system measured on the lobe of the molecule.* ***f***. *TERS spectrum of the FePc/NaCl/Ag(111) system measured on the center of the molecule.*

11. Density functional theory calculations of minimum energy configurations

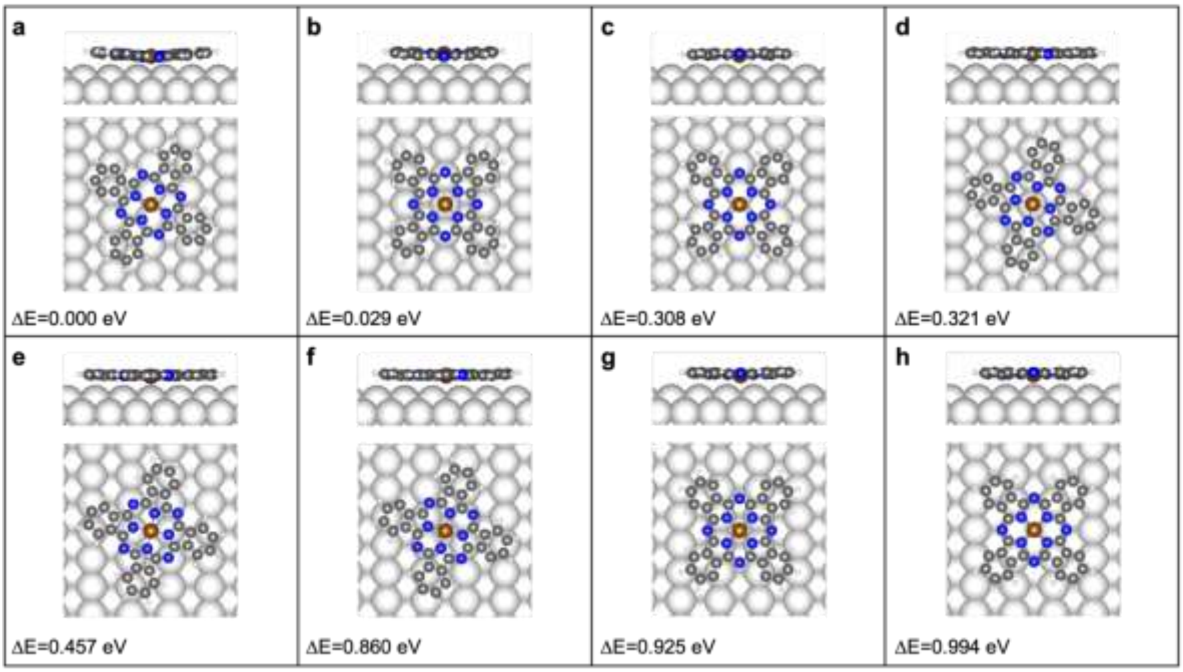

Figure S12. *DFT optimized structures of FePc on Ag(110) and their relative energies with respect to the most stable conformation. The main characteristics of the calculations are listed in table S1. Experimentally we observe the two lowest energy configurations corresponding to the a) and b) panels.*

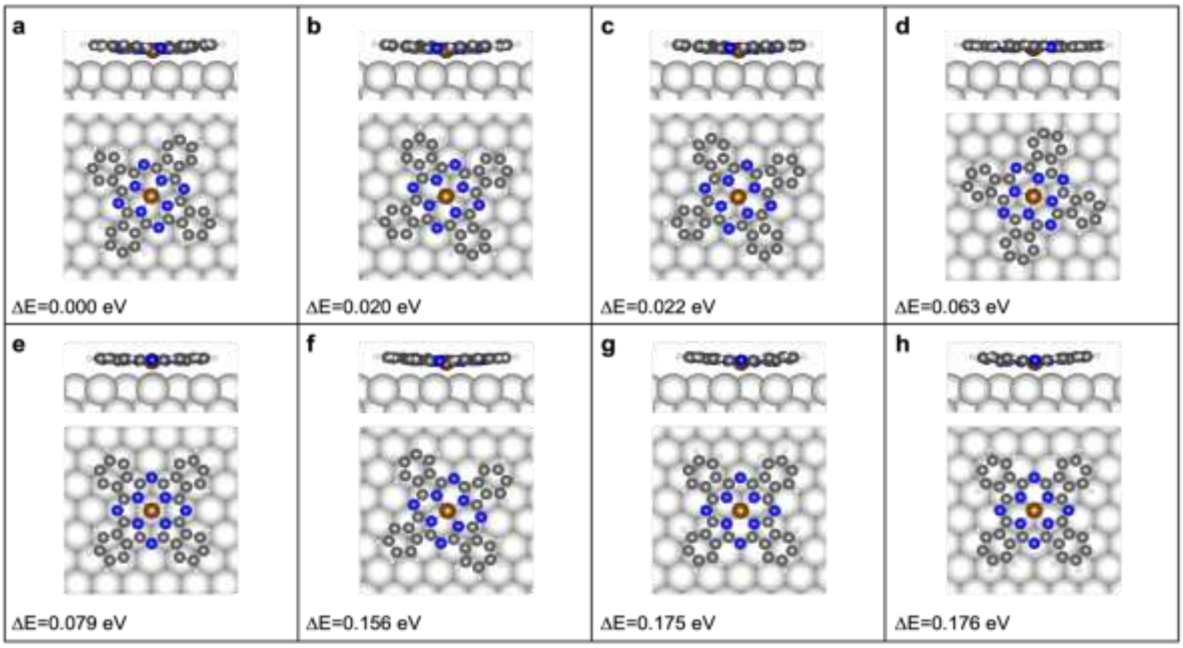

Figure S13. *DFT optimized structures of FePc on Ag(111) and their relative energies with respect to the most stable conformation. The main characteristics of the calculations are listed in table S1. Experimentally we observe the lowest energy configuration corresponding to the a) panel.*

12. Density functional theory analysis of charge transfer in FePc/Ag interfaces

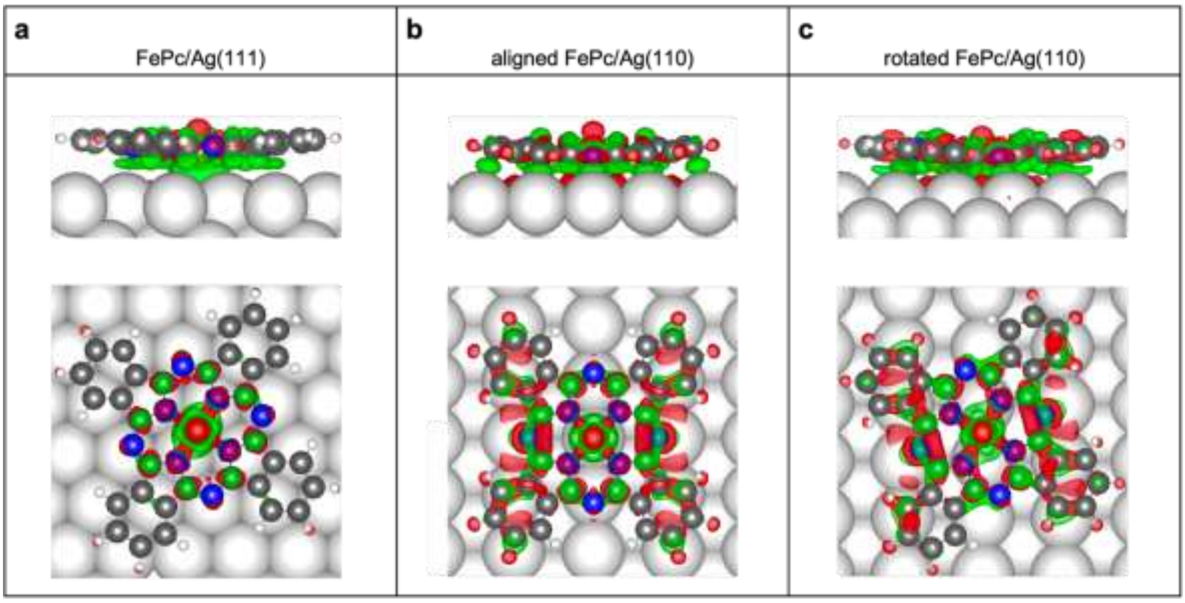

Figure S14. *Distribution of the charge transfer upon adsorption of FePc/Ag(111) (left), aligned FePc/Ag(110) (center) and rotated FePc/Ag(110) (right). Green and red colors denote regions where there is an increase or a decrease of the electron density, respectively.*

	**FePc/Ag(111)**	**aligned FePc/Ag(110)**	**rotated FePc/Ag(110)**
Fe	0.92	0.92	0.91
N_i	-1.12	-1.12	-1.09
N_o	-1.21	-1.25	-1.23
C_i	0.94	0.96	0.93
C_r	-0.04	-0.05	-0.04
H	0.07	0.08	0.06
**Q_{tot}**	**-0.81**	**-0.80**	**-0.82**

Table S1: *Atomic charges of FePc in the three experimentally observed structures and their total charges. N_i denotes the inner nitrogen atoms (directly bonded to Fe), N_o denotes the outer nitrogen atoms (not bonded to Fe), C_i refers to the inner carbon atoms (bonded to N), and C_r to the carbons of the aromatic rings. Reported values correspond to the average charge for each type of atom.*

13. Supporting references

41. Cirera, B. et al. Upgrade of a variable temperature scanning tunneling microscope for nanometer-scale spectromicroscopy. MethodsX **14**, 103156 (2025).

42. Liu, S., Wolf, M. & Kumagai, T. Plasmon-Assisted Resonant Electron Tunneling in a Scanning Tunneling Microscope Junction. Phys. Rev. Lett. **121**, 226802 (2018).

43. Kresse, G. & Furthmüller, J. Efficient iterative schemes for ab initio total-energy calculations using a plane-wave basis set. Phys. Rev. B **54**, 11169–11186 (1996).

44. Román-Pérez, G. & Soler, J. M. Efficient Implementation of a van der Waals Density Functional: Application to Double-Wall Carbon Nanotubes. Phys. Rev. Lett. **103**, 096102 (2009).

45. Dion, M., Rydberg, H., Schröder, E., Langreth, D. C. & Lundqvist, B. I. Van der Waals Density Functional for General Geometries. Phys. Rev. Lett. **92**, 246401 (2004)

46. Perdew, J. P., Burke, K. & Ernzerhof, M. Generalized Gradient Approximation Made Simple. Phys. Rev. Lett. **77**, 3865–3868 (1996).

47. Grimme, S., Antony, J., Ehrlich, S. & Krieg, H. A consistent and accurate ab initio parametrization of density functional dispersion correction (DFT-D) for the 94 elements H-Pu. J. Chem. Phys. **132**, 154104 (2010)

48. Grimme, S., Ehrlich, S. & Goerigk, L. Effect of the damping function in dispersion corrected density functional theory. J. Comput. Chem. **32**, 1456–1465 (2011).

49. Dunning, T.H. Gaussian basis sets for use in correlated molecular calculations. I. The atoms boron through neon and hydrogen. J. Chem. Phys. **90**, 1007 (1989)

50. Kesharwani M. K., Brauer B., Martin J. M. L., J. Phys. Chem. A **119,** 1701, (2015)
